# Light-Sensing Properties of Amorphous Vanadium Oxide Films Prepared by RF Sputtering

**DOI:** 10.3390/s23041759

**Published:** 2023-02-04

**Authors:** Rodica Plugaru, Iuliana Mihalache, Cosmin Romaniţan, Florin Comanescu, Silviu Vulpe, Gabriel Craciun, Neculai Plugaru, Nikolay Djourelov

**Affiliations:** 1 National Institute for Research and Development in Microtechnologies-IMT Bucharest, Erou Iancu Nicolae 126 A, 077190 Voluntari, Ilfov, Romania; 2 Extreme Light Infrastructure-Nuclear Physics (ELI-NP), “Horia Hulubei” National R&D Institute for Physics and Nuclear Engineering (IFIN-HH), 077125 Magurele, Romania

**Keywords:** light-sensing, vanadium oxide, RF sputtering

## Abstract

In this study we analyzed the structure and light-sensing properties of as-deposited vanadium oxide thin films, prepared by RF sputtering in different Ar:O_2_ flow rate conditions, at low temperature (e.g., 65 °C). X-ray diffraction (XRD), Scanning Electron Microscopy (SEM-EDX), Raman spectroscopy and X-ray photoelectron spectroscopy (XPS) were employed to analyze the film microstructure, composition and the oxidation states of vanadium ions. The SEM micrographs evidence V_x_O_y_ films with smooth surfaces, whereas the XRD patterns show their amorphous structure. Raman spectra indicate an increased structural disorder in the films deposited in Ar:O_2_ flow comparatively with those deposited solely in Ar flow. The XPS data suggest the modification of the oxidation state from V^4+^ to V^5+^, thus proving the formation of the V_2_O_5_ phase when increasing the oxygen content, which further affects the films’ optical properties. We observed a good stability of the photogenerated current in Si/SiO_2_/V_x_O_y_/TiN heterostructures upon excitation with pulses of UV (360 nm), VIS (white light) and NIR (860 nm) light. The responsivity, detectivity and linear dynamic range parameters increase with the O/V ratio in the V_x_O_y_ films, reaching comparable values with photodetectors based on crystalline V_2_O_5_ or VO_2_.

## 1. Introduction

Vanadium oxides have gathered a constantly growing interest in different fields of applications, due to their ability to tune material properties according to the vanadium oxidation state. Vanadium can occur in numerous oxidation states, e.g., V^5+^, V^4+^ or V^3+^, which is advantageous for the preparation of thin films with different electrical and chemical properties for a wide range of applications [1]. Several studies have been conducted, and a rich phenomenology has been revealed in crystalline compounds, such as VO, VO_2_, V_2_O_3_ or V_2_O_5_ oxides. For instance, V_x_O_y_ compounds can change their optical properties due to external stimuli in the form of photon radiation (photochromic) [2], change in temperature (thermochromic) [3] and voltage pulse (electrochromic) [4]. More specifically, thin films of VO_2_ and V_2_O_3_ have been found to show good thermochromism in the infrared region, while maintaining transparency to visible light, e.g., a smart window modulates infrared irradiation from a low-temperature transparent state to a high-temperature opaque state. Vanadium sesquioxide (V_2_O_3_) is a member of vanadium oxides (V_x_O_y_) called Magneli phases, defined by the general stoichiometric formula V_n_O_2n−1_ [5]. Their property of undergoing a metal-insulator transition (MIT) with the change in temperature has attracted thermo- and electrochromic applications, such as smart windows [6], ultra-fast nanoelectronic switches [7], transistors [8], thermoelectric devices [9]. Moreover, vanadium pentoxide (V_2_O_5_) has proved its ability for electrochromic and charge storage devices, due to its ability for Li-ion intercalation in the layered crystal structure [10,11,12,13]. On the other hand, amorphous vanadium oxides (a-V_x_O_y_) were relatively unexplored in comparison with the crystalline counterparts. These films became popular mostly for energy-related applications. To date, a-V_x_O_y_ has been obtained by electrochemical oxidation [14], reactive sputtering [15], using a combination of sol-gel processing paired with electrochemical deposition [16], atomic layer chemical vapor deposition [17], atomic layer deposition [18], or recently, gas impulse magnetron sputtering [19].

Magnetron sputtering seems to be one of the most interesting processing routes of V_x_O_y_ thin films, because of the possibility of producing large area thin films with repeatable properties, a good uniformity of deposition, as well as a good adherence to various substrates. Also, the possibility to control the process parameters allows the preselection of the films’ physical properties at the stage of synthesis, enabling a rational design of the future opto-electronic devices. From the applications point of view, it was shown that the a-V_x_O_y_ films with a thickness of around 650 nm can be used for Li- and Na-ion batteries [17], while other studies presented the applications of composites, such as vanadium oxide on graphene nanosheets [18] for stable, high energy lithium-ion anodes, a-V_x_O_y_/C composites for positive electrodes in rechargeable aluminum batteries [19] or a-VO_x_/MXene nanohybrid architecture for high-performance Na-ion batteries [20]. Recently, the applicability of a-VO_x_ for hydrogen gas sensing applications has been demonstrated, with the films’ conductivity controlled from p- to n-type via increasing the amount of the oxygen in the gas impulse magnetron sputtering process [21]. The investigations of the VO_x_ films deposited onto quartz substrate by RF sputtering deposition from a vanadium target at 400 °C substrate temperature, with 8.5% O:Ar ratio, showed that amorphous films have flat and stable optical transmittance curves. Films with different thicknesses could be used to control the intensity of the light [22]. Moreover, it was shown that the amorphous V_2_O_5_ films provided a quick response time (4.9 ms) with high detectivity (1.45 × 10^12^ Jones) for broadband transparent photodetectors [23].

In this study we investigate the relationship between the deposition Ar:O_2_ flow rate and the thin films’ structure and stoichiometry, pointing out that the increased oxygen content in the deposition process leads to a transition from the VO_2_ to the V_2_O_5_ phase, which further affects the light-sensing properties of V_x_O_y_ thin films. Also, we show that the responsivity, detectivity and linear dynamic range parameters of the heterostructures with different V_x_O_y_ amorphous films increase with the O/V ratio, reaching comparable values with recently published results for photodetectors based on crystalline VO_2_ [24] or V_2_O_5_ [25].

## 2. Materials and Methods

The V_x_O_y_ films were deposited on Si (100) n-type (1–10 Ω·cm)/SiO_2_ and glass substrates by RF sputtering, using a V_2_O_3_ target (99.99% purity, Testbourne Ltd., Basingstoke Hampshire, UK). The deposition was performed in Ar or Ar:O_2_ plasma with the temperature of the substrate maintained at 65 °C. The pressure inside the deposition chamber was 5 mTorr and the RF power was 300 W. The V_x_O_y_ films were prepared by changing the Ar:O_2_ ratio in the deposition plasma. We used three working conditions: (A) Ar (30 sccm), (B) Ar (30 sccm): O_2_ (0.5 sccm) and (C) Ar (30 sccm): O_2_ (1 sccm) mass flow rate. Electrical contacts on the film surface were obtained by deposition of titanium nitride (TiN) square pads, with different areas, S (1) = 9 mm^2^, S (2) = 1 mm^2^, S (3) = 0.25 mm^2^ and S (4) = 0.09 mm^2^, through a mechanical mask. The deposition process was performed by RF sputtering from a Ti target, in atmosphere of Ar (20 sccm): N_2_ (1 sccm), at a pressure of 5 mTorr. All the deposition processes were performed by using a PlasmaLab System 400 (Oxford Instruments Plasma Technology, North End Yatton Bristol, UK) equipment. The structure, morphology and composition of the films were analyzed by XRD and SEM-EDX, using a SmartLab/X-ray Thin Film Diffraction System (Rigaku Corporation, Tokyo, Japan) and a Nova Nano SEM 630 Field Emission Gun Scanning Electron Microscope (FEG-SEM), equipped with EDX spectroscopy (FEI Company, Hillsboro, OR, USA), respectively. Raman spectra of the as-deposited films were acquired by using a High-Resolution Raman Spectrometer-LabRAM HR 800 with a 633 nm HeNe Laser (Horiba, Jobin Yvon, France). X-ray photoemission spectroscopy (XPS) spectra were recorded on a Sigma Surface Science photoelectron spectrometer (Scienta Omicron, Taunusstein, Germany) equipped with a 160 mm hemispherical energy analyzer with a 1D detector (ASPECT) and using an Al Kα X-ray source at 13 kV at a power of 200 W. The analysis area was 1.3 × 1.3 mm^2^. The transmission and absorption analysis were performed at room temperature using Cary 5000 UV-Vis-NIR Spectrophotometer (Agilent Technologies, Santa Clara, CA, USA). The electrical characteristics of the Si/SiO_2_/V_x_O_y_/TiN heterostructures were measured using SCS 4200 Keithely (Tektronix Inc. Beaverton, Oregon, USA)—Suss Microtech system (Cascade Microtech, Beaverton, OR, USA), at 15 V, in the dark and under illumination with white light (10 mW/cm^2^) and monocromatic wavelengths in the UV (360 nm) and NIR (860 nm) ranges, using a set-up with laser diode sources with intensity of 140 mW/cm^2^.

## 3. Results

### 3.1. Morpho-Structural Investigations

**Figure 1:**
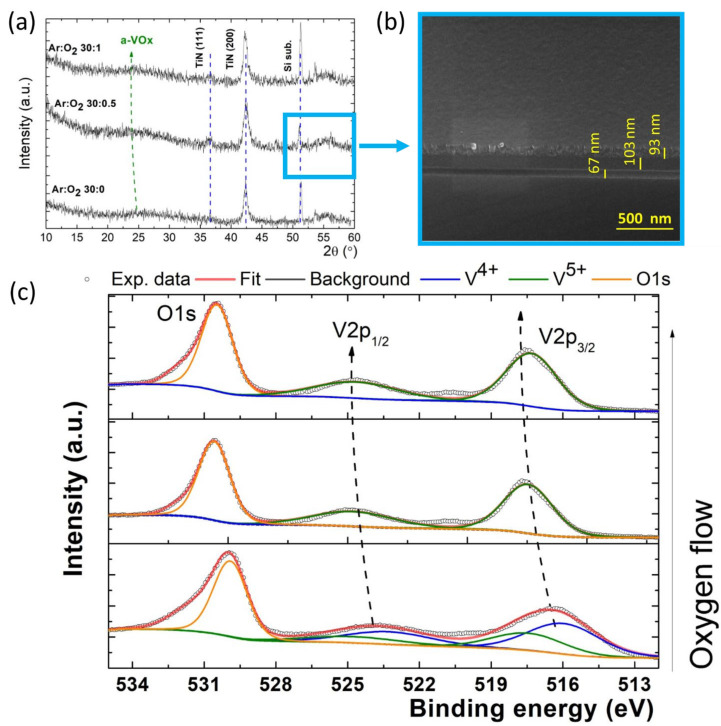
(**a**) The XRD patterns of the heterostructures with vanadium V_x_O_y_ films deposited in different Ar:O_2_ flux conditions. (**b**) Cross-view SEM micrograph of a Si/SiO_2_/V_x_O_y_/TiN heterostructure with V_x_O_y_ film obtained in Ar (30 sccm): O_2_ (0.5 sccm) plasma. (**c**) The XPS fit on the V2p and O1s signal.

In order to assess the impact of the Ar:O_2_ gas flow rate on the crystalline structure of the V_x_O_y,_ thin films, the XRD patterns were recorded at the grazing-incidence. The diffraction patterns of the Si/SiO_2_/V_x_O_y_/TiN heterostructures are shown in Figure 1a.

Figure 1b presents a SEM micrograph of the Si/SiO_2_/V_x_O_y_/TiN heterostructure with V_x_O_y_ film deposited in Ar (30 sccm):O_2_ (0.5 sccm) gas flow rate conditions, process (B). The SEM micrograph shows the presence of a 67 nm thick bottom SiO_2_ film, a 109 nm thick V_x_O_y_ film and a 93 nm thick top film of TiN. All the V_x_O_y_ films exhibit smooth surfaces (no roughness) with no specific features, as also observed by SEM plan view investigation.

The XRD patterns also reveal the presence of the cubic TiN film with a = 0.423 nm, confirmed by the TiN (111) and TiN (002) reflections at 36.72 and 42.27° (ICDD card no. 031-1403). In addition, a sharp diffraction peak from the Si substrate occurred at 51.21°. Moreover, the broad diffraction feature located at smaller angles (e.g., between 20 and 32°) shows the amorphous nature of the V_x_O_y_ films, regardless of the oxygen flow in the deposition process. That indicates that, at the low temperature of the substrate (~65 °C), the deposited material may be just sticking to the Si/SiO_2_ substrate surface at its place of hitting, with almost no surface diffusion. The substrate temperature is not high enough to provide sufficient thermal energy to the ad-atoms to find the locations for further bonding, resulting in an amorphous structure [26].

Previous XRD studies showed that the deposition of the crystalline V_2_O_5_ phase occurs at around 200 °C in the case of d.c. reactive magnetron sputtering [27]. Recent investigations on V_2_O_5_ RF sputtered films also showed that sputtered V_2_O_5_ films grown at room temperature have an amorphous structure [28]. Other investigations on the V_x_O_y_ films deposited by pulsed laser deposition (PLD), showed that the amorphous–crystalline transition temperature of V_2_O_5_ films is around 200 °C [26,29], also suggesting that the fundamental thermodynamic parameter, deposition temperature, governs the crystallinity of the samples. However, in our experiment, it could be observed that the XRD broad halo is shifted to smaller angles with increasing oxygen flow, which can be attributed to an additional interplanar spacing, induced in the presence of the oxygen.

The XPS investigation was carried out on as-grown samples on the glass substrates, in order to obtain more information on the chemical state of the vanadium species and for additional phase identification. The O 1s and V 2p signals were recorded together in one energy window. These spectra reveal the presence of V 2p doublets, as well as the O 1s core level peak, and the fitting was performed using a mixed Lorentzian–Gaussian function, see Figure 1c. Whereas the peak of O 1s was found at 530.4 eV, the V2 p_3/2_ and V2 p_1/2_ present shift to higher binding energy (BE), when the oxygen flow increases, namely from 523.8 eV to 524.7 eV and from 516.4 eV to 517.6 eV, respectively. The BE values for vanadium oxides are in a good agreement with the previous work on VO_x_ [30,31]. The slight shifts in the peak position were further ascribed to the modification of the oxidation, evolving from a V^4+^ oxidation state to V^5+^ (black arrow was used to show this trend). Interestingly, it is important to observe a similar trend in the XRD patterns (see blue dashed arrow), indicating a different crystal packing and an additional strain in the presence of oxygen. The latter observation could give the explanation of the different band gaps of the films, as will be shown later. According to the XPS data, this trend implies a transition from a VO_2_ phase to V_2_O_5_ phase, as the oxygen flow increases. Additional confirmation of the V_2_O_5_ presence at high oxygen flow is given by the spin-orbit splitting of approximately 7.5 eV between V2 p_3/2_ and V2 p_1/2_ orbitals, as well as by the energy difference of 12.9 eV between the binding energy of V2 p_3/2_ and the O 1s orbital [16,32].

**Figure 2:**
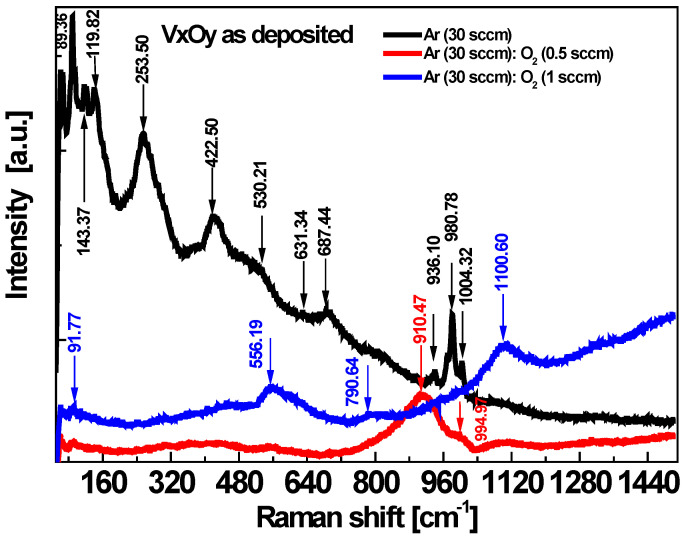
Raman spectra of the as−deposited V_x_O_y_ films.

Moreover, the Raman spectra of the films illustrate the significant influence of the oxygen flow in the Raman modes, see Figure 2. The peaks are more intense and well resolved in the spectrum of the film deposited in the Ar flow and become broader in the spectra of the films where an oxygen flow is added in the process.

The Raman spectrum of the film obtained in process (A) shows several sharp intense maxima located in the 89.36–143.37 cm^−1^ region, as well as at 253.50 cm^−1^, 422.50 cm^−1^, 530.21 cm^−1^, 631.34 cm^−1^, 687.44 cm^−1^, 936.10 cm^−1^, 980.78 cm^−1^ and 1004.32, while the Raman spectrum of the film obtained in process (B) presents only a low intensity, broad peak centered at 910.47 cm^−1^ with a shoulder at 994.97 cm^−1^. The Raman spectrum of the film obtained in the richest atmosphere of O_2_, i.e., process (C), exhibits broad and weak peaks positioned at 556.19 cm^−1^, 790.64 cm^−1^ and 1100.60 cm^−1^, respectively. The major vibrational region in the Raman spectra of vanadium oxide systems is situated in the 100–1200 cm^−1^ range and covers: V−0 terminal stretching that occurs at 770–1050 cm^−1^, the V−0−V stretching region at 500–800 cm^−1^ and the bending mode at 150–400 cm^−1^. Lattice vibrations of crystalline compounds may also be present below 150 cm^−1^ [33].

The Raman spectra are very sensitive to vanadium oxygen coordination; the analysis of the specific position of the Raman bands in comparison with the reference compounds could give an insight towards their structural assignment. As such, the Raman data could reveal the effect of the oxygen flow in the deposition process on the structure of the V_x_O_y_ films. The Raman spectra of vanadium oxide thin films deposited on Si by reactive sputtering, by varying the O_2_/Ar gas flow ratio, e.g., 1.5/100 sccm, 1.5/50 sccm, show main peaks at ~225 cm^−1^ and 504 cm^−1^, corresponding to the V_2_O_3_ phase, while a 1.3/100 sccm gas flow ratio leads to the formation of a VO_2_ phase. The further increase in the oxygen content in the deposition process determines a transition to the V_2_O_5_ phase, with the characteristic Raman peaks at 145 cm^−1^, 195 cm^−1^, 284 cm^−1^, 303 cm^−1^, 405 cm^−1^, 483 cm^−1^, 701 cm^−1^ and 992 cm^−1^ [34].

In reference [15], the maxima observed in the Raman spectrum of a V_2_O_5_ film deposited by RF sputtering in an amorphous state are attributed to the stretching modes of the V3–O bonds (520 cm^−1^), V2–O (650 cm^−1^) and to the stretching mode of terminal oxygen atoms V^5+^=O (1027 cm^−1^). Also, a maximum situated at 932 cm^−1^ is attributed to V^4+^=O bonds. Shiver et al. reported Raman peaks in the Raman spectra of vanadium oxides without long range order, situated at 950 cm^−1^ related to V^4+^=O bonds and, respectively, at 1020 cm^−1^ related to V^5+^=O [35]. The dominant bands in films B) and C) suggest the V^4+^ and, respectively, V^5+^ valence states in agreement with the XRD and XPS data. The broad and weak Raman bands corresponding to films B) and C) indicate the presence of a high degree of amorphous phase.

The morphology and structural investigations show that RF sputtering from a V_2_O_3_ target led to a more crystalline structure of the films deposited in process (A) comparatively with the (B) and (C) processes. Different oxygen flow added to the deposition atmosphere beside Ar flow led to different microstructural characteristics. The effect on optical properties is further investigated by absorption and transmission measurements.

### 3.2. Absorption and Transmission Measurements on VxOy Films

**Figure 3:**
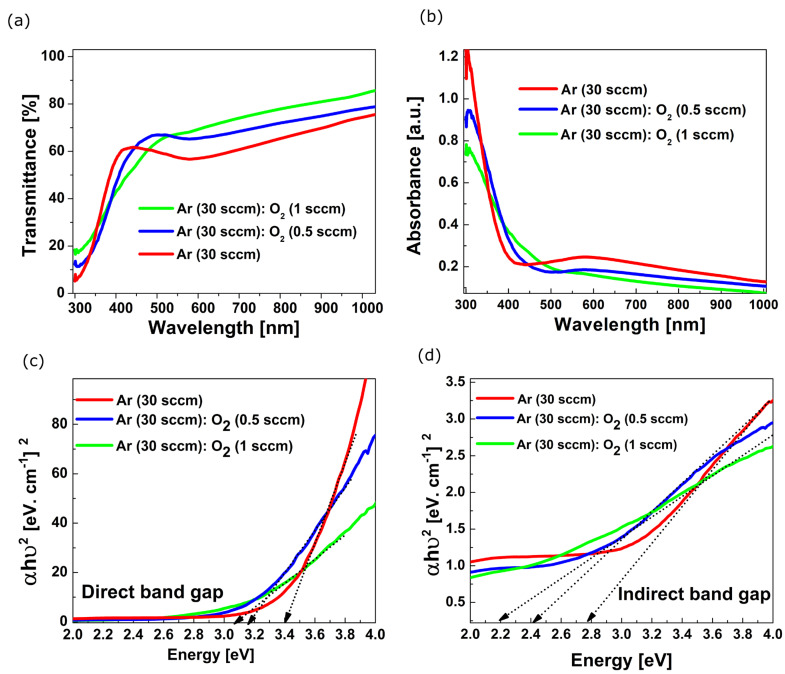
Transmission (**a**) and absorption (**b**) graphs of the films deposited at different concentrations Ar:O_2_. Direct (**c**) and indirect (**d**) band gap calculation.

The transmission and absorption graphs of the V_x_O_y_ films deposited on glass substrates are shown in Figure 3a,b.

The V_x_O_y_ films have transmission values of: (A) 57%, (B) 65% and (C) 68%, respectively (at 570 nm), which indicates a better transmission of the films deposited in a rich oxygen atmosphere. The red shift of the transmission edge is also observed. The absorption curves indicate a decrease in the optical band gap of these films. The Eg_optic_ values were calculated from the absorption spectra using the Tauc equation [36]:

$$\left(\alpha h\nu \right)\propto {\left(h\nu -E_{gopt}\right)}^{n}\;$$

where hν is the incident photon energy, α the absorption coefficient and the exponent *n* = 1/2, 2 for direct allowed transition and indirect allowed transitions, respectively. Both direct and indirect Eg_optic_ decrease with the increase in the oxygen content in the films, as one may observe in Figure 3a,b. The allowed direct and indirect Eg_optic_ values are listed in Table 1.

One may observe that the optical band gap energy, Eg_opt_, in both direct and indirect cases, shifts towards lower energy with the increase in oxygen concentration. According to reference [37], the presence of the tetravalent vanadium ion (V^4+^) enhances the UV light absorption in the oxides. Since the XPS data indicated V^4+^ is suppressed at high oxygen flow, this could be the explanation for the band gap energy decreasing.

### 3.3. Electrical I−V and C−V Characteristics

**Figure 4:**
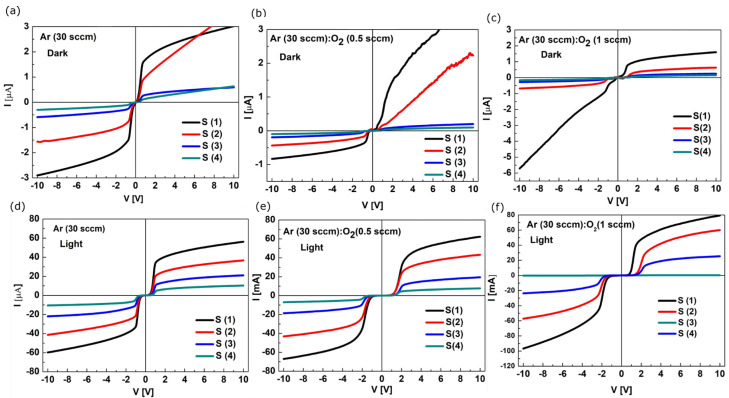
The I−V characteristics of the V_x_O_y_ films with various oxygen content. The contact area is a parameter. Measurements performed in conditions of dark (**a**−**c**) and under white light illumination (**d**−**f**).

The current-voltage (I-V) characteristics of the Si/SiO_2_/V_x_O_y_/TiN heterostructures measured on the two front contacts with areas of: S (1) = 9 mm^2^, S (2) = 1 mm^2^, S (3) = 0.25 mm^2^ and S (4) = 0.09 mm^2^, spaced at 0.2 mm, are presented below in Figure 4a−f.

The current-voltage (I−V) characteristics show the behavior of two Schottky diodes in configuration, “back−to−back”. The intensity of the dark current measured on the S1 contacts at 2 V is 2 µA in the (A) type heterostructure, see Figure 4a, and 1 µA in the (C) type heterostructure, see Figure 4c. The I-V characteristics of the (B) type heterostructure do not show saturation at positive polarization (0 ±10 V), as can be seen in Figure 4b. The dark current intensity at 2 V is 1.5 µA. For all the heterostructures, the intensity of the current measured under white light illumination (10 mW/cm^2^) increases by about an order of magnitude comparatively to the intensity of the dark current. The values of the photogenerated current are (A) 40 µA, (B) 30 µA and (C) 48 µA, respectively, see Figure 4d−f. The turn on voltage of the Schottky diode, V_on_, shifts toward positive values as the oxygen content in the films increases, suggesting the increase in the density of TiN/V_x_O_y_ interface states.

**Figure 5:**
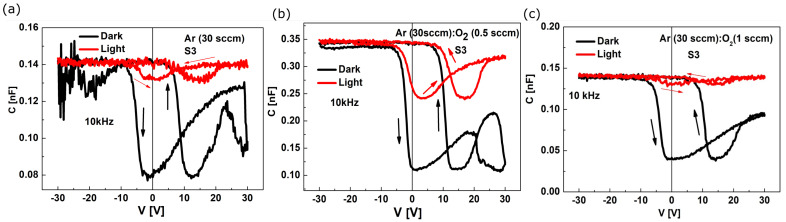
The C−V characteristics of the Si/SiO_2_/V_x_O_y_/TiN heterostructures with V_x_O_y_ films obtained in (**a**), (**b**) and (**c**) conditions (**a**–**c**). Measurements were performed for heterostructures with S3 = 0.25 mm^2^, at 10 kHz, in conditions of dark (black line) and under white light (red line) illumination. Arrows in the graphs indicate the voltage sweep direction.

The capacitance-voltage (C−V) characteristics of the heterostructures with the films deposited in the A), B) and C) conditions are shown in Figure 5a–c. The measurements were performed in the “front to back” configuration of the heterostructures with the contact area of S (3) = 0.25 mm^2^, in dark and under white light illumination. The hysteresis behavior could be observed in the C−V characteristics measured under forward and reverse voltage sweep in the −30 V to 30 V range. The flat band voltage (V_FB_) values, determined from dark C−V measurements by extending the linear region in the plot of 1/C^2^ to the voltage axis [38], vary from 7.3 V (deposition process A) to 9.6 V (deposition process B) and 10.2 V (deposition process C). The positive shift of V_FB_ indicates the charge trapping (electrons) characteristics that are related to the gradual increase in the oxidation state from V^4+^ to V^5+^ in V_x_O_y_ as−grown films.

### 3.4. Current−Time Characteristics

**Figure 6:**
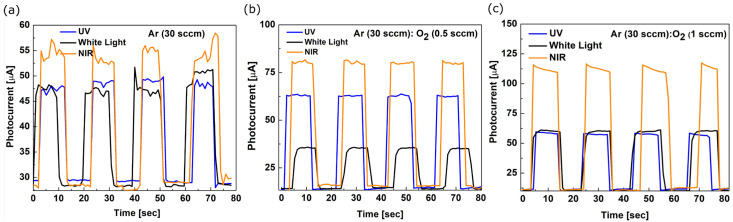
Photogenerated currents, measured by applying pulses of (**a**) UV (360 nm), (**b**) VIS (white light) and (**c**) NIR (860 nm) light.

The photogeneration processes under illumination with various wavelengths were investigated aiming to evaluate the sensing properties of amorphous vanadium oxide thin films obtained by (A), (B) and (C) conditions. The current-time, (I−t), characteristics presented in Figure 6a−c show the currents photogenerated in Si/SiO_2_/V_x_O_y_/TiN heterostructures, under excitation with different wavelengths.

The films demonstrate the good stability of the photogenerated currents upon excitation with light pulses. The intensity of the photogenerated current by applying pulses lasting 10 s varies depending on the wavelength. The characteristics indicate that the films are responsive in UV, VIS and NIR spectral domains. However, the selectivity changes with the O/V ratio and the film stoichiometry. The I−t characteristics presented in Figure 6b indicate increased sensitivity in the NIR as well as UV range for the V_x_O_y_ films prepared in process (B), whereas the I−t characteristics presented in Figure 6c indicate increased sensitivity only in the NIR range for the films prepared in process (C), for which the photogenerated current has double intensity. All the V_x_O_y_ films exhibit increased responsivity to NIR light, with the photogenerated current values of 53 µA, 80 µA and 110 µA, respectively. Previous studies attributed the near-infrared absorption in V_2_O_5_ films to small polaron effects [39]. Kim et al. [23] reported that amorphous V_2_O_5_ thin film transparent photodetector yields an excellent performance with quick response times of 4.9 ms for blue, 12.7 ms for green and 16.1 ms for red light, respectively.

The main parameters used for photodetection characterization are Responsivity (R), specific detectivity (D*) and the linear dynamic range (LDR). Responsivity can be described as:

(1) $$R=\frac{J_{ph}}{P_{in}}\;$$

which is the ratio of the output current density (*J_ph_*) to the power of input illumination (*P_in_*) and indicates how sensitive a sensor is to the light.

Specific detectivity (D*) indicates the ability to detect a weak light signal and can be calculated with the following equation:

(2) $$D^{*}=\frac{R}{\sqrt[{\;}]{2\;q\;J_{d}}}$$

where R is the responsivity, *q* is the electron charge and *J_d_* is the dark current density.

Finally, linear dynamic range (LDR) describes the response range in which the photodetector sensitivity is linear with the input light:

(3) $$LDR=20\log\;{\left.\left(\frac{J_{ph}}{J_{d}}\right.\right)}^{\;}$$

**Figure 7:**
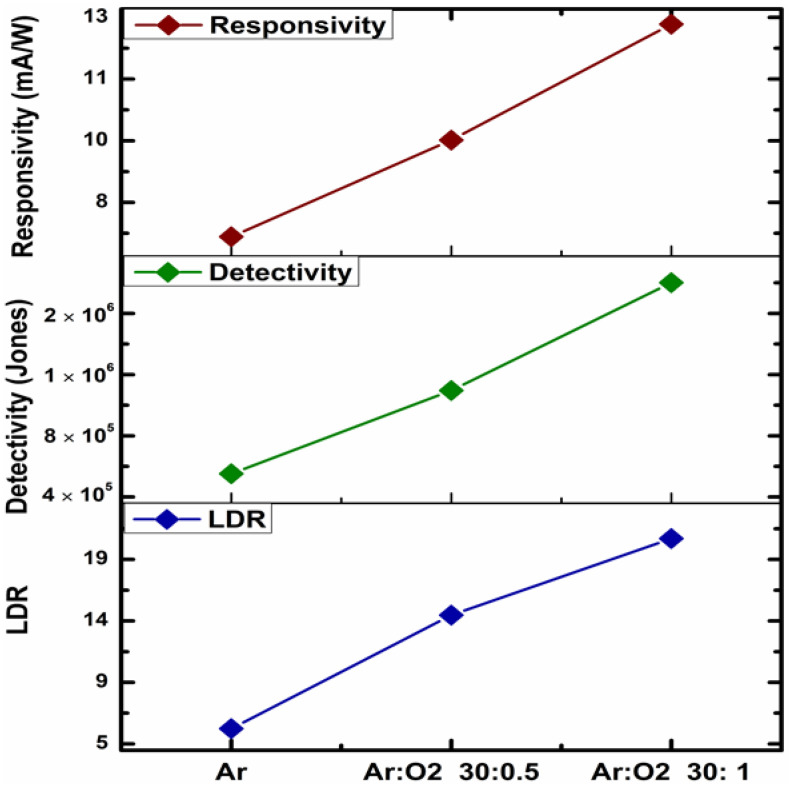
Responsivity, detectivity and linear dynamic range calculated for V_x_O_y_ film-based heterostructures exposed to NIR wavelength (860 nm).

Figure 7 shows the values of R, D* and LDR parameters calculated for Si/SiO_2_/V_x_O_y_/TiN heterostructures exposed to NIR illumination where the highest sensitivity was previously demonstrated. All the parameters increase proportionally with the phase transitioning triggered by increased oxygen flow during the deposition of V_x_O_y_ thin film. The heterostructure based on the film obtained in the richest O_2_ atmosphere achieved the maximum values of R~13 mA/W, D*~1.7 × 10^6^ Jones and LDR~20 dB, respectively. The responsivity range is similar with other reports on V_x_O_y_ materials [24,25,40] and makes the heterostructures compelling for NIR photodetection applications [41]; however, high voltage bias is also required. The observed dark current reached a value of 1 mA leading to decreased detectivity. The level of defect-related recombination is presumably high in the as−deposited amorphous vanadium oxide structures; however, the further optimization of Ar:O_2_ flow deposition could enable reduced charge recombination followed by charge transport improvement.

## 4. Conclusions

We investigated the effect of Ar:O_2_ mass flow rate in the RF magnetron sputtering process of V_x_O_y_ film deposition at low temperatures, by means of spectroscopic XRD, XPS and Raman techniques. It was evidenced that the increase in oxygen content determines a vanadium oxidation state transition from V^4+^ (VO_2_) to V^5+^ (V_2_O_5_). According to the absorption and transmission measurements on V_x_O_y_ films, different microstructural features as resulted in the deposition process significantly affect the optical band gap energy and the films’ light-sensing properties. The Si/SiO_2_/V_x_O_y_/TiN heterostructures demonstrated a broad spectral sensitivity. The responsivity, detectivity and the dynamic linear response of amorphous V_x_O_y_ thin film photodetectors were evaluated for NIR monochromatic light. The highest responsivity was achieved for the films deposited in the Ar (30 sccm): O_2_ (1 sccm) mass flow rate condition. The main parameters of photodetectors based on amorphous vanadium thin films could be comparable to the previously reported results for crystalline VO_x_−based photodetectors.

## Figures and Tables

**Table 1:** The optical band gap Eg_opt_ of the films.

Film Deposition Process	Eg_opt_ Direct [eV]	Eg_opt_ Indirect [eV]
(A) Ar (30 sccm)	3.37	2.76
(B) Ar (30 sccm):O_2_ (0.5 sccm)	3.16	2.39
(C) Ar (30 sccm):O_2_ (1 sccm)	3.08	2.19

## Data Availability

Not applicable.
